# Visualization and analysis of peripheral drift illusion

**DOI:** 10.1186/1471-2202-12-S1-P347

**Published:** 2011-07-18

**Authors:** Keiichiro Inagaki, Shiro Usui

**Affiliations:** 1 RIKEN, Computational Science Research Program, 2-1, Hirosawa, Wako-shi, Saitama 351-0198, Japan; 2 RIKEN Brain Science Institute (BSI), 2-1, Hirosawa, Wako-shi, Saitama 351-0198, Japan

## 

The peripheral drift illusion (e.g. Rotating snake [1]) yields rotating motion on our peripheral vision. It was reported that the order of different four luminance regions is essential for the illusion [1]. Moreover, Conway et al. have suggested that luminance or contrast dependent latency in response of V1/MT direction selective cells for those kinds of luminance (black, dark-gray (blue), light-gray (yellow), and white) was contributed on perception of the rotating illusory motion [2]. In the present work, we modeled V1 and MT as a retinotopic map using those direction selective cells [2] and investigated whether this model can reproduce the rotating illusory motion.

## Models

The model of V1 and MT were constructed as a retinotopic map of those direction selective cells. The temporal responses of V1 and MT direction selective cells were estimated from the Conway’s single unit data [2] using regression analysis and were parameterized for either contrast or luminance. In the parameterization for the luminance, the estimated neuronal responses were normalized to take a range from 0.0 (black) to 1.0 (white). In ones for the contrast, low and high contrast were defined to be negative and positive value, and estimated responses were normalized to take range from 0 (gray) to 1.0 (white) or -1.0 (black).

## Simulation results

We used Rotating snake stimulus for a model input. In the simulation, the input stimulus image was divided into four luminance regions (black, dark gray (blue), light gray (yellow), and white); the neuronal responses were separately computed in each region. In agreement with experimental study [1], the similar rotating motions were observed in the transition of neuronal responses of the V1 and MT model for contrast; any experimentally consistent rotating motions were not found in those for luminance. For the control stimulus that does not evoke the rotating illusion, any rotation motions were not observed. We also analyzed those reproduced responses by optical flow [3]. The flows corresponding to rotating illusory motion were reproduced in peripheral region of the map of V1 and MT for contrast, but ambiguous flows were observed in those for luminance. For the control stimulus, the flows for clockwise and counter clockwise rotation were detected, and those seem to cancel the perception of rotating illusion.

## Conclusion

In the present study, we modeled the V1 and MT direction selective cells based on the single unit data, and created the retinotopic map of V1 and MT. The models successfully reproduced the similar rotating motions observed in the experimental study [1]. By analyzing the V1 and MT responses transition by optical flow, the flows for the rotating illusory motion were represented peripherally in those maps reconstructed based on contrast, while the flows countervailing the perception of rotating illusion were observed in the control stimulus. The results suggested that the signal source for the perception of rotating snake was represented in both V1 and MT, and the contrast is a key signal to drive an illusory motions in peripheral drift illusion as reported by previous studies [1,2].

## References

[B1] Kitaoka A Ashida H Phenomenal characteristics of the peripheral drift illusion VISION 2003 15 261 262

[B2] Conway RB Kitaoka A Yazdanbakhsh A Pack CC Livingstone MS Neural basis for a powerful static motion illusion J Neurosci 25 5651 5656 15944393 10.1523/JNEUROSCI.1084-05.2005 PMC1431688

[B3] Lucas B Kanade T An Iterative Image Registration Technique with an Application to Stereo Vision Proceedings of the Seventh International Joint Conference on Artificial Intelligence (IJCAI) 1981 674 679

